# Etymologia: *Prototheca*

**DOI:** 10.3201/eid2711.211554

**Published:** 2021-11

**Authors:** Rüdiger D. Ollhoff, Fábio P. Sellera, Fabio C. Pogliani

**Affiliations:** Pontifícia Universidade Católica do Paraná, Curitiba, Brazil (R.D. Ollhoff);; Universidade de São Paulo, São Paulo, Brazil (F.P. Sellera, F.C. Pogliani);; Universidade Metropolitana de Santos, Santos, Brazil (F.P. Sellera)

**Keywords:** Prototheca, protothecosis, algae, pathogenic algae, fungal-like organisms, saprophytes, zoonoses, Wilhelm Krüger, Friedrich Wilhelm Zopf

## *Prototheca* [pro″to-the′kə] 

**Figure 1:**
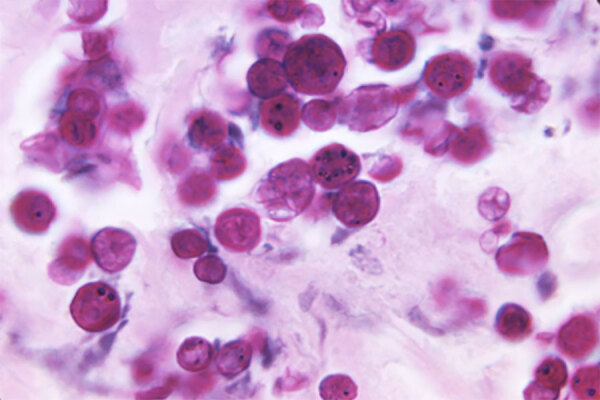
Periodic acid‒Schiff‒stained tissue sample from a case-patient who had protothecosis, showing several sphere-like cells of *Prototheca* spp. Source: Dr. Jerrold Kaplan, Centers for Disease Control, 1971.

**Figure 2:**
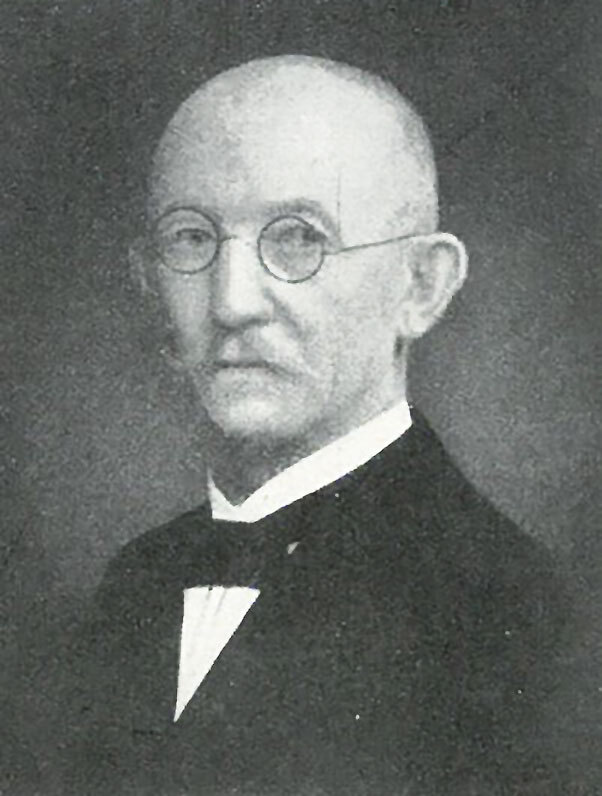
Wilhelm Krüger (1857‒1947). Source: Institute for Sugar Beet Research (http://www.ifz-goettingen.de).

From the Greek *proto-* (first) + *thēkē* (sheath), *Prototheca* is a genus of variably shaped spherical cells of achloric algae in the family *Chlorellaceae* (Figure 1). Wilhelm Krüger, a German expert in plant physiology and sugar production, reported *Prototheca* microorganisms in 1894, shortly after spending 7 years in Java studying sugarcane (Figure 2). He isolated *Prototheca* species from the sap of 3 tree species. Krüger named these organisms as *P. moriformis* and *P. zopfii*, the second name as a tribute to Friedrich Wilhelm Zopf, a renowned botanist, mycologist, and lichenologist.

Protothecosis affects humans and wild and domestic animals, primarily causing mastitis in cows. Human protothecosis was reported in 1964 from a skin lesion in a farmer from Sierra Leone. There are increasing reports of infections in immunocompromised patients. Debates regarding *Prototheca* taxonomy persist.
